# Proceedings of the 2017 International Conference of Precision Health and Precision Nutrition: Shanghai, China. 6-7 November 2017

**DOI:** 10.1186/s12986-018-0261-3

**Published:** 2018-05-09

## Session I Big cohorts, big data, and precision health

### S1 Mendelian randomization, molecular mediation to understand and prioritize for interactive targets in populations of diverse ethnicities

#### Simin Liu^1,2^

##### ^1^Brown University School of Public Health, Providence, RI, USA; ^2^Alpert School of Medicine, Providence, RI, USA

One fundamental principle of biology is interaction, understanding of which forms the basis for the development of novel therapeutic and preventive strategies with proven clinical efficacy (Phase III) and population effectiveness (Phase IV and implementation). The existing research and implementation processes for both drug therapy and new diagnostic tools are very fragmented and grossly insufficient. First, the drug development process takes too long (10-15 years/drug) and cost too much (~$1 billion/drug) with high failure rates (~80%) even for targets and compounds that survive through Phases II development. Second, the lack of assessment for interactions among genetic factors and therapeutic agents in almost all human studies may be a rate-limiting step for success (or lead to post-marketing failure due to as yet unknown off-target detrimental effect). Third, current structure does not encourage basic scientists to work closely with clinical and population scientists. Last and most importantly, insufficient measurement of covariates often precludes from confidently predicting intermediate or long-term effects of various agents.

I propose here that by applying innovative systems biology approaches to existing cohort resources, the biological pathways and gene networks that are perturbed by genetic variations and their interactions with potential targets and phenotypic risks could be modeled in both the preclinical and post-marketing period to evaluate potential benefits and risks associated with both new drugs and/or targets. We have recently used this unique and comprehensive approach in assessing biological targets in relation to risk of developing type 2 diabetes (T2D) and cardiovascular complications among T2D patients in diverse ethnic populations. Herein I use several examples to demonstrate that such a strategy should be adopted in the drug (including nutritional supplements) development process to enhance the probability of discovery and validation of targets, thus reducing wasteful resources. To complement this unbiased approach, we will also focus the sex-steroid pathway recently identified including sex-hormone binding globulin (SHBG) as therapeutic targets for enhancing or impairing insulin sensitivity, metabolic syndrome and type 2 diabetes risk particularly concerning multiple germline mutations in the sex-hormone pathways as well as the SHBG gene to be predictive of both metabolic syndrome and T2D risk in multiple cohorts of men and women.

### S2 Study for precision medicine of metabolic diseases

#### Guang Ning^1,2,3^

##### ^1^National Clinical Research Center for Metabolic Diseases, Changsha, Hunan, China; ^2^Rui-Jin Hospital, Shanghai Jiao Tong University School of Medicine, Shanghai, China; ^3^Shanghai Institute of Endocrine and Metabolic Diseases, Shanghai, China

The consistently increased morbidity of chronic non-communicable diseases, especially the metabolic diseases is becoming a major impediment for public health; the diversified etiologies and the complicated additive-effects bring on great difficulties for prevention and treatment. On one hand, external factors like lifestyle, dietary structure, gut microbiota, and environmental contamination exacerbate the prevalence of metabolic disease; on the other hand, internal factors including genetic variation, family history, racial background, and basal metabolic rate determine the susceptible population; these two parts interact with each other and appear to be constantly dynamic. Consequently, the traditional research strategies with monocenter and univariate on single population would constrain the development and progress for study of metabolic diseases. For this reason, promoting multicentral, successive, dynamic, and multi-population based methods including the deep metabolic phenotype analysis, collection of comprehensive environmental factors, dynamic surveillance for lifestyle, construction for multi-omics system, massive deep-sequencing, and combined validation for biological functions, are expected to depict the traits of formation and evolution of metabolic diseases for individuals as well as the whole population in a more accurate and comprehensive way, and would certainly become a novel orientation of precise study for metabolic diseases in the future.

### S3 Realising the power of prospective biobanks in diverse populations

#### Zhengming Chen

##### Nuffield Department of Population Health, University of Oxford, Oxford, UK

Chronic diseases, such as stroke, heart disease, cancer and diabetes, are the leading causes of disability and death worldwide. Despite recent advances, our ability to prevent and treat these conditions is still limited. Understanding what causes these diseases in diverse populations with different lifestyles, environments and genetic architectures can lead to improved disease prevention and risk prediction, and the development of “precision medicine”. Unique opportunities to fulfill these goals are offered by prospective “biobank” studies, with detailed characterization of large numbers of apparently healthy individuals from the general population, using conventional and novel technologies, and with electronic monitoring of their health status.

In the last decade, many large prospective biobank studies of global significance (e.g. US PMI cohort, UK Biobank) are being or have been assembled. China Kadoorie Biobank (CKB) is one of the world’s largest studies of this kind, involving 512,891 adults recruited during 2004-08 from 10 diverse areas in China, with extensive data collected at baseline and periodic resurveys, on lifestyle, environmental, and physiological factors, and with long-term storage of biological samples. To date, >0.5 million fatal and non-fatal disease events of >1000 different types (e.g. stroke, heart disease, cancer, diabetes, fracture, cataract and rheumatoid arthritis) have been recorded among participants. These exposure and health outcome data are now being complemented by blood assays of genetic (e.g. 20 million variants), metabolomic (e.g. ~1000 metabolites), proteomic (e.g. ~400 inflammation and other biomarkers) and infective biomarkers, with many novel findings starting to emerge. The uniquely powerful and rich resources in CKB and other big biobanks will enable scientists to make many important discoveries relevant to risk prediction, disease prevention and treatment, benefiting populations worldwide.

## Session II Systems epidemiology approaches to metabolic health

### S4 Recent advances in precision nutrition and cardiometabolic diseases

#### Frank B. Hu

##### Department of Nutrition, Harvard T.H. Chan School of Public Health, Boston, MA, USA

Recent advances in powerful tools such as genomics, metabolomics, and gut microbiome have offered new opportunities as well as challenges in the application of precision nutrition for the prevention and management of type 2 diabetes (T2D). The integration of such technologies into epidemiological studies, referred to as “systems epidemiology”, can enhance our understanding of biological mechanisms underlying diet and human health. This approach can also enable us to achieve better assessment of diet and nutritional status in free-living populations by identifying novel biomarkers of dietary intakes. Nutritional genomics has identified genetic variants that influence intakes and metabolism of specific nutrients and predict individuals’ variability in response to dietary interventions. Metabolomics has revealed metabolomic fingerprints of food and nutrient consumption and has uncovered new metabolic pathways that are potentially modified by diet. In addition, dietary interventions have been shown to alter abundance, composition, and activity of gut microbiota that are relevant for food metabolism and glycemic control. By integrating these technologies with big data analytics, precision nutrition has the potential to provide personalized nutrition guidance to achieve more effective T2D prevention and management. Despite recent advances, major challenges still exist, including non-replication of study results, difficulty in translation of research findings into practice, and high cost. Although commercial companies have promoted personalized nutrition assessment and genetic testing, there is little evidence on the benefits of these approaches for improving diet and preventing disease. Therefore, it is essential to balance the investment in precision nutrition, which targets individual characteristics, with public health nutrition, which aims to improve the health of populations.

### S5 Big data and precision medicine

#### Jiarui Wu

##### Key Laboratory of Systems Biology, Chinese Academy of Sciences, Shanghai, China

Facing the grand challenges of complex diseases such as cancer and diabetes, researchers recently have developed “Precision Medicine” to identify new biomarkers for prediction of disease progression and accurate diagnosis of individual. Discovery of biomarkers by “Precision Medicine” relies on two new approaches to analyze big data. Firstly, the approach for integrating big data derived from genomics, proteomics, metabolomics and other omics, for integrating big data from molecular level to physiological and pathological levels. Secondly, the approach for building individual-centric biomedical database that consists of multilayered and highly interconnected biological parameters, and then construct a knowledge network of biomedical research, which could be used for new taxonomic classification of diseases.

### S6 A gut microbiota perspective of precision health and nutrition

#### Xun Xu^1,2^, Junhua Li^1,2^, Huanzi Zhong^1,2,4^, Huijue Jia^1,2^, Zhuye Jie^1,2^, Ruixin Liu^3^, Yanyun Gu^3^, Karsten Kristiansen^1,2,4^

##### ^1^BGI-Shenzhen, Shenzhen, China; ^2^China National GeneBank, BGI-Shenzhen, Shenzhen, China; ^3^State Key Laboratory of Medical Genomes, National Clinical Research Center for Endocrine and Metabolic Diseases, Ruijin Hospital, Shanghai Jiao Tong University School of Medicine, Shanghai, China; ^4^Laboratory of Genomics and Molecular Biomedicine, Department of Biology, University of Copenhagen, Copenhagen, Denmark

Diet, particularly macronutrients, have a major influence in shaping gut microbiome both in the short and long term, which in turn can impact metabolic heath. The comprehensive integrated gene catalog of human gut microbiota has greatly advanced metagenome-wide association studies (MGWAS) on the interactions between host-nutrition-microbiome in modulating metabolic diseases. MGWAS have enhanced our understanding of the dysbiosis of gut microbial composition and function in relation to type 2 diabetes and atherosclerotic cardiovascular disease. Further, MGWAS incorporated with mouse studies have identified novel links between gut microbial species and host metabolites in the development of obesity, pointing to potential microbial targets for intervention.

People with interindividual variability of their gut microbiome have experienced vastly heterogeneous metabolic responses to dietary factors and oral medicines. These can be mediated by several microbial-derived metabolites, such as secondary bile acids, short chain fatty acids and trimethylamine N-oxide. A recent study has convincingly linked the bile acids based microbiota-host interactions to the host metabolic benefits of antidiabetic agents.

Importantly, to pave the way for microbiota-focused precision medicine and nutrition, efforts on collection and analyses of individuality multi-omics data, including the gut metagenomics, human genetics, metabolomics and diet records, are required to establish their causal relationships in people who respond beneficially to a given intervention and therapy.

### S7 Integration of multi-omics data to identify biomarkers for cancer risk

#### Wei Zheng

##### Division of Epidemiology at Vanderbilt University Medical Center, Nashville, TN, USA

Over the past 10 years, through the use of large-scale genomic data, genome-wide association studies have discovered approximately 10,000 genetic loci associated with human diseases and other complex traits. In addition to genomics, other omic technologies have also been used increasingly in epidemiologic studies, providing tremendous opportunities to study causes of diseases, identify disease biomarkers, and understand biological mechanisms for disease development and progression. Over the years, in collaboration with investigators from many other institutions, we have initiated large studies and research consortia to interrogate the whole genome, transcriptome, metabolome, and epigenome to identify biomarkers for risk of cancer and other chronic diseases. In large genome-wide association studies that currently include approximately 180,000 participants of Asian descent recruited in nearly 50 studies conducted in mainland China, Hong Kong, Taiwan, Korea, Japan, Malaysia, Singapore, Thailand, and the USA, we have discovered more than 50 novel genetic susceptibility risk loci for breast and colorectal cancer, type 2 diabetes, and obesity. Using metabolomics tools, we have identified multiple new biomarkers for risk of colorectal and pancreatic cancers and type 2 diabetes. Recently, we conducted a series of studies integrating transcriptomic and genomic data to systematically search the transcriptome to uncover genes associated with the risk of breast, prostate, colorectal, and ovarian cancers, resulting in the identification of a large number of novel associations. For example, in a large breast cancer study including approximately 119,000 cases and 101,000 controls included in the Breast Cancer Association Consortium, by integrating genomic and transcriptomic data, we found nearly 100 genes showing a significant association with breast cancer risk after adjusting for multiple comparisons. This study demonstrated the potential of integrating multi-omics data to identify biomarkers for cancer risk. Proper use of multi-omic data will undoubtedly improve the understanding of the etiology of human diseases and accelerate the pace of discovery of the causes of diseases and identification of disease biomarkers, leading to the design of cost-efficient prevention strategies.

**Acknowledgments**

We receive NIH funding for our research and our research has been approved by IRB at Vanderbilt University Medical Center.

### S8 Linking of omics-based biomarkers with nutrition and cardiometabolic health in Chinese

#### Liang Sun^1,2^, Yiwei Ma^1,2^, Huaixing Li^1,2^, Xu Lin^1,2^

##### ^1^Shanghai Institutes for Biological Sciences, Chinese Academy of Sciences (CAS), Shanghai, China; ^2^Key Laboratory of Nutrition and Metabolism, CAS, Shanghai, China

With rapid development of advanced technology, omics-based biomarkers are expected to provide a more precise tool to predict and control diseases, and also to evaluate nutrition status. However, limited prospective studies have systematically investigated major nutritional and genetic factors for cardiometabolic diseases in Chinese with the world largest populations of obesity and type 2 diabetics (T2D). Thus, by utilizing multiple omics approaches in a 6-yr population-based cohort study and also leading a national genome-wide association study (GWAS) for T2D in Chinese Hans, over 100 genetic variants were identified or confirmed to be associated with obesity, T2D and hypertension. Moreover, about 50 genetic variants were documented to influence fatty acids, vitamin D and iron metabolic pathways. Meanwhile, multiple nutritional biomarkers (high carb or dairy intakes and iron, vitamin D, vitamin B1 status) were showed to be associated with prevalence or incident of metabolic syndrome (MetS), T2D and other health outcomes. For instance, high carbohydrate dietary pattern appeared to promote levels of 16:1n-7 and other fatty acids from de novo lipogenesis (DNL) pathway. Increased levels of 16:1n-7 fatty acids were associated with a higher 6-year incidence of MetS and T2D. In our recent GWAS for monounsaturated fatty acids, GCKR-rs1260326 and PKD2L1-rs603424 were linked with altered 16:1n-7 and other fatty acid levels by modifying DNL pathway. Moreover, a panel of acylcarnitines, especially long-chain acylcarnitines, was showed to substantially improve predictive ability for incident diabetes beyond conventional risks. There were about 70% participants in our cohort population having vitamin D deficiency and low plasma 25(OH)D significantly associated with high MetS risks. In our recent randomized, double-blinded controlled trial, daily supplementation with 2000 IU vitamin D3 (the upper intake level in China) for 20 weeks significantly raised 25(OH)D concentrations, but still left 25% participants with uncorrected deficiency. Genetic factors exerted stronger impact than non-genetic factors (baseline value, BMI and gender) on 25(OH)D responses; while overweight persons showed lower 25(OH)D response than that in normal weight persons. Overall, our studies provide important insights regarding roles of genetic and nutrient-related biomarkers on metabolic diseases and nutritional status in Chinese and these findings are useful for achieving strategies of precision nutrition.

**Acknowledgements**

The study was supported by the Ministry of Science and Technology of China (2017YFC0909700, 2016YFC1304903) and Chinese Academy of Sciences (ZDBS-SSW-DQC-02).

### S9 Advanced quantitative metabolomics tools for molecular epidemiology

#### Hehua Lei^1^, Fuhua Hao^1^, Hong Lin^3^, Yulan Wang^1,4^, and Huiru Tang^2^

##### ^1^Wuhan Institute of Physics and Mathematics, Chinese Academy of Sciences, Wuhan, China; ^2^State Key Laboratory of Genetic Engineering, Zhongshan Hospital and School of Life Sciences, Human Phenome Institute, Fudan University, Shanghai International Centre for Molecular Phenomics, Collaborative Innovation Center for Genetics and Development, Shanghai, China; ^3^Shanghai Metabolome Institute (Wuhan) Ltd, Wuhan, China; ^4^Collaborative Innovation Center for Diagnosis and Treatment of Infectious Diseases, Zhejiang University, Hangzhou, China

Metabonomes of mammals contain tens of thousands different types of metabolites with molecular mass smaller than 3000 Daltons. They have many important functions and a huge concentration dynamic ranges, diverse physicochemical properties, matrices and are originated from both endogenous and exogenous sources [1-3]. Therefore, quantitative metabonomic analysis is essential for understanding the molecular aspects of mammalian physiology and pathophysiology of various diseases. The combined nuclear magnetic resonance (NMR) and mass spectra (MS) methods have become increasingly attractive with complementary metabolomic information. In this presentation, we will discuss the requirements of quantitative metabonomics and strategies to fulfill such tasks followed with some recent advances in our lab in sensitive and efficient quantification of metabolites. These include metabolites containing amino groups cover dozens pathways [4] and play vital roles in redox homeostasis and biosyntheses of proteins, nucleotides and neurotransmitters. Quantitative metabolic fluxomics approaches will also be reported in the context of the stress-induced metabolomics responses [5]. Many more methods will further be discussed for ultrasensitive quantification of sterols and bile acids, eicosanoids and acyl carnitines. These methods will be potentially useful for molecular epidemiological studies of many different biological events.

**Acknowledgements**

We acknowledge the National Key R&D Program of China (2017YFC0906800) and National Natural Science Foundation of China (91439102, 81590953) for financial supports.

**Reference**s

[1] Nicholson G, Rantalainen M, Maher A D, et al. Human metabolic profiles are stably controlled by genetic and environmental variation. Molecular systems biology, 2011, 7(1): 525.

[2] Tang H, Wang Y. Metabonomics: a revolution in progress. Prog Biochem Biophys 2006, 33(5): 401-417.

[3] Teague C, Holmes E, Maibaum E, et al. Ethyl glucoside in human urine following dietary exposure: detection by 1H NMR spectroscopy as a result of metabonomic screening of humans. Analyst, 2004, 129(3): 259-264.

[4] Wang J, Zhou L, Lei H, et al. Simultaneous quantification of amino metabolites in multiple metabolic pathways using ultra-high performance liquid chromatography with tandem-mass spectrometry. Scientific reports, 2017, 7(1): 1423.

[5] Wan Q, Wang Y, Tang H. Quantitative 13C Traces of Glucose Fate in Hepatitis B Virus-Infected Hepatocytes. Analytical chemistry, 2017, 89(6): 3293-3299.

### S10 How can systems approaches add value to nutrition and metabolism?

#### Lisa Krämer^1^, Jean-Pierre Trezzi^3^, Jochen Schneider^3^, Karsten Hiller^1,2^

##### ^1^Department for Bioinformatics and Biochemistry, BRICS, Technische Universität Braunschweig, Braunschweig, Germany; ^2^Helmholtz Zentrum für Infektionsforschung, Braunschweig, Germany; ^3^Luxembourg Centre for Systems Biomedicine, University of Luxembourg, Luxembourg

Uptake and metabolism of food is a highly complex process and requires the coordinated interaction of cells, tissues and organs in multi-cellular organisms to establish a metabolic homeostasis. Already minor off-tunes in this interaction can cause disease or malfunction of the whole organism. On the other hand, the organism has to be prepared to act on various fuel types and thus to convert various types of nutrients into energy and biomass as well as building up a storage pool for times of fasting. To ensure the immediate and stable availability of energy even under high load conditions where complete carbohydrate oxidation is too slow or even not possible, the body must ensure that production and clearance of metabolites such as glucose, lactic acid or alanine are balanced.

Here we demonstrate how systems approaches can be applied to quantitatively monitor these metabolic processes on a whole organism scale. Stable-isotope labeling is a safe procedure to follow the metabolism of pathway metabolites and their turn-over rates in vivo. We established a sensitive procedure to accurately determine isotopic enrichment patterns in human plasma after oral ingestion of a 13C labeled glucose tracer. The time-resolved sampling is based on mass spectrometry and dried blood spot sampling. The obtained data is then incorporated into a specific tailored ODE based metabolic model of the Cori cycle. By solving the equations of this system, we could determine quantitative and robust values for glucose production (GP) and gluconeogenesis (GNG) rates for each studied subject.

While already providing exact and quantitative metabolic turn-over rates for metabolites directly involved in glucose metabolism, such a “small system” does not highlight other parts of whole body homeostasis as for example metabolism of amino acids. To address this limitation, we are extending our focus and include quantitative flux data for more metabolites. For that purpose, we follow the fate of fully 13C labeled wheat flour after ingestion by human individuals. Due to the fact that all carbon atoms in this wheat are substituted by 13C isotopes, every wheat molecule has an increased molecular mass. The major components of wheat flour are starch and protein and the digestion and metabolism of these bio-polymers can be traced by determining isotopic enrichment patterns in plasma metabolites.

**Acknowledgements**

Funding for this project is provided by Unilever R&D Vlaardingen, The Netherlands.

### S11 Integrating genetic, genomic and environmental data to define the mechanistic basis of type 2 diabetes

#### Mark McCarthy^1,2,3^

##### ^1^Oxford Centre for Diabetes, Endocrinology and Metabolism, University of Oxford, Churchill Hospital, Old Road, Headington, Oxford, UK; ^2^Wellcome Trust Centre for Human Genetics, University of Oxford, Roosevelt Drive, Oxford, UK; ^3^Oxford NIHR Biomedical Research Centre, Churchill Hospital, Old Road, Headington, Oxford, UK

The growing prevalence of type 2 diabetes (T2D) highlights the limitations of available preventative options, and high rates of diabetes complications attest to the inadequacies of current treatments. Novel therapeutic strategies need to be informed by a more complete understanding of the molecular and physiological basis of disease, delivering validated interventional targets and biomarkers to define disease risk, progression, and subtype.

My group, working within large global consortia, uses human genetics to deliver this understanding. Growing availability of exome sequence and array data now delivers coding variant associations that can plug directly into functional studies. However, the main repository of variant association for T2D remains the ~150 common variant signals uncovered by genome-wide association study (GWAS), most of which map outside coding sequence. We are implementing a multifaceted approach that combines genome-scale and focused functional studies to unlock the biology within these loci.

We use fine-mapping to improve localisation of causal variants, and map these onto regulatory annotations from key tissues, most notably the human islet. This provides a platform for identifying downstream transcripts through tissue-specific cis-eQTL analyses and conformational capture. We combine these “regulatory variant” data with transcript level information to define the best-supported transcripts in each GWAS region. Finally, we connect loci through analyses of protein-protein interaction, co-expression and pathway data. These efforts are starting to bear fruit, with around one-third of GWAS signals now featuring a well-supported priority transcript. We follow up these priority candidates through cellular, molecular, rodent and human studies to consolidate mechanistic evidence, and to integrate additional non genetic risk factors. Finally, we use the biological insights derived from these studies to support efforts at translation through (a) identification of novel therapeutic targets; (b) stratification of disease risk and phenotype; (c) biomarker discovery; (d) therapeutic optimization; (e) more precise specification of complication risk; and (f) causal inference regarding environmental risk factors.

**Acknowledgements**

The study was funded by Wellcome, Medical Research Council, EU (Horizon 2020), Innovative Medicines Initiative, National Institute for Health Research, National Institutes of Health, Juvenile Diabetes Research Foundation.

## Session III Personalized nutrition for disease prevention

### S12 Fatty acids and carbohydrates in healthful dietary patterns to prevent cardiovascular disease

#### Frank Sacks

##### Department of Nutrition, Harvard School of Public Health, Boston, MA, USA

I discuss the evidence from clinical trials, prospective observational studies, and mechanistic studies that link dietary fats and carbohydrates to cardiovascular disease (CVD). This discussion is in large part derived from an American Heart Association Presidential Advisory on Dietary Fats and CVD. In summary, randomized trials that replaced saturated fat with polyunsaturated fat, mainly linoleic acid, lowered risk of CVD. The higher the quality of the individual trial, the greater the reduction in CVD. Meta-analysis of four, high quality, core trials found reduction in CVD of about 30%. Other meta-analyses that included trials that had serious problems in design or execution also found significant reduction in CVD but about 20% rather than 30%, as would be expected. The greater the reduction in saturated fat the greater the reduction in risk of CVD. In contrast, a meta-analysis of six trials that replaced saturated fat or total fat with carbohydrate did not show significant reduction in CVD. This indicates that any evaluation of a macronutrient needs to consider the replacement.

These findings in randomized trials are supported by prospective observational studies that found that replacement of saturated fat with polyunsaturated fat at 5% of total daily energy intake was associated with a 25% lower risk of CVD; replacement with monounsaturated fat was associated with 15% lower risk. In contrast, replacing saturated fat with carbohydrate was not associated with reduced risk. Thus, the results of observational studies and randomized trials are closely aligned. Recently, meta-analyses on dietary fat have been published, reporting that saturated fat is not associated with CVD. However, these analyses did not take into account whether the saturated fat is replaced with unsaturated fat or carbohydrate --- a crucial distinction.

The causal connection between dietary saturated fat and CVD and protection by polyunsaturated fat is supported by atherosclerosis studies in non-human primate experiments, and by effects on LDL-cholesterol.

In summary, I present evidence that is clear and convincing that to lower risk of CVD dietary intake of saturated fats should be replaced with unsaturated fats, especially polyunsaturated fats.

**References**

[1] Sacks F M, Lichtenstein A H, Wu J H Y, et al. Dietary fats and cardiovascular disease: a presidential advisory from the American Heart Association. Circulation, 2017, 136(3): e1-e23.

### S13 Soy food consumption and breast cancer risk and prognosis

#### Xiao-Ou Shu

##### Vanderbilt University Epidemiology Center and Vanderbilt University Medical Center, Nashville, TN, USA

Soy food is a rich source of isoflavones, a group of phytoestrogens that have both estrogen-like and estrogen-antagonistic effects. Isoflavones have been shown to: increase the synthesis of sex-hormone-binding globulin, lower the biological availability of sex hormones; inhibit 17β-hydroxysteroid dehydrogenases, thereby reducing estrogen synthesis; and increase clearance of steroids from the circulation. In addition, isoflavones have several non-hormone-mediated, anti-cancer properties. Over the last 3 decades, epidemiological studies conducted among Asian women, including our studies conducted among Chinese women in Shanghai, have consistently shown soy food intake to be inversely associated with breast cancer risk, whereas studies conducted among non-Asian women have largely generated null results. The importance of early-life or lifetime exposure to soy food on breast health was first suggested by our study of Chinese women and confirmed subsequently by two US studies and a cohort study that we conducted among Chinese women. The estrogen-like property of soy isoflavones, on the other hand, had been a concern for breast cancer patients. Using data from a longitudinal study conducted among Chinese women in Shanghai, we showed that soy food intake was inversely associated with the risk of cancer recurrence and mortality. These results were confirmed by a pooled analysis of four cohort studies that included over 9,500 breast cancer patients. These findings resulted in a change in dietary recommendations which conclude that soy food intake is safe for breast cancer survivors. Our subsequent studies found that high soy food intake was positively associated with menopausal symptoms and inversely associated with bone density among breast cancer survivors, suggesting that soy food acts as an estrogen antagonist rather than estrogen agonist among breast cancer patients. Our recent study found that long-term soy food consumption was associated with increased expression of tumor-suppressor miRNAs and genes, and decreased expression of oncogenes, especially cell-growth genes, in triple negative breast cancer tumor tissue, and was positively associated with circulating level of B-lymphocytes among healthy individuals. These findings shed light on the biological mechanisms by which soy food consumption influences breast cancer risk and/or prognosis.

### S14 Precision medicine in diabetes diagnosis and treatment - a clinician’s perspective

#### Linong Ji

##### Department of Endocrinology and Metabolism, Peking University People’s Hospital, Beijing, China

From a clinician’s perspective, precision medicine in vast majority of diabetes patients is still a dream. Diabetes is a heterogeneous disease with very complex interaction between genetic and environmental factors. The current diagnosis of diabetes is basing on the blood glucose cutoffs closely related to increased risk for chronic diabetic complications but not hyperglycemia itself. The natural history of diabetes and risk for complications show great variation among individuals due to the different causes and pathogenesis of diabetes. For example, glucokinase gene variants induced diabetes patients (MODY2) usually present with mild non-progressive hyperglycemia and low risk for microvascular disease because their HbA1c levels seldom rise beyond 7.6%, and HNF1A variants induced diabetic patients (MODY3) have relatively high risk for chronic complications because of gradually impaired beta cell function and severe hyperglycemia. In the past decade, the genome wide association study and whole exome sequencing have revealed more than one hundred genetic variants associated with diabetes and its related traits, of which a limited number of genes are demonstrated to involve in insulin secretion and insulin sensitivity, most genes were not well known on their functions. That is to say, we know so little on diabetes that we could not finely and correctly type diabetes, the first step for precision medicine of diabetes. In fact, a minority of monogenic diabetes patients have benefited from individualized treatment, suggesting the causes and pathophysiology of diabetes are key factors for precision medicine. What’s more, through studies on monogenic diabetes, we have understood more and more biologic signal pathways involved in the development of diabetes, but the detailed crosstalk between these pathways have not been well known. Therefore, basing on the facts that there is susceptibility gene overlap among diabetes, hyperglycemia, obesity and other diabetes-related traits, for patients with type 2 diabetes, we can guess that the numbers and the kinds of pathways and their contributions would determine when they develop diabetes, how fast their diabetes progress, what features they present and what hypoglycemia agents fit them. On the other hand, it is stressed that we should identify the biomarkers for impaired pathways and subsequently select appropriate drugs, so the findings from monogenic disease in diabetes related pathways would be helpful to screen these biomarkers and guide precision medicine. Although many antidiabetic drugs have been developed, they only targeted limited pathways. Thus, a limited kind of drugs is another obstacle for precision medicine of diabetes even though we could precisely type diabetes. In summary, in the present time, because of our limitation in understanding the complexity and heterogeneity of diabetes and availability of antidiabetic agents, only a small fraction of patients could benefit from precision medicine. In the future, identifying the new biological target of a drug and developing new hypoglycemic agents is necessary in order that more and more patients are provided with precision medicine.

### S15 Precision medicine in the diabetes prevention and management

#### Cheng Hu

##### Shanghai Diabetes Institute, Shanghai Key Laboratory of Diabetes Mellitus, Shanghai Clinical Center for Endocrine and Metabolic Diseases, Shanghai Jiao Tong University Affiliated Sixth People’s Hospital, Shanghai, China

Type 2 diabetes mellitus (T2D) is a metabolic disease characterized by hyperglycemia with complex etiology. There are about 100 million adults with diabetes in China. Long term hyperglycemia may cause multiple organ injuries including stroke, blindness, myocardial infarction, renal failure and amputation. So far, the research and development of early warning and individualized treatment of diabetes is still a worldwide problem. Genetic markers can provide early warning information before obesity, abnormal glucose regulation and other clinical phenotypes appear. Therefore, we aim to analyze the genetic etiology of T2D and investigate early genetic markers for the occurrence and development of T2D in Chinese population for the guidance of clinical prevention and individualized treatment.

We carried out large scale genetic researches of T2D in Chinese Han population. We validated 28 European-derived T2D susceptible loci and identified 12 novel susceptible genes in Chinese. Moreover, we constructed the genetic risk score model for T2D based on the 40 genetic loci previously identified in Chinese population and reported the predictive value of genetic model in prospective cohorts. The construction of the model is of great value to reveal the etiology and early warning of T2D in Chinese.

In addition, we performed the pharmacogenomics studies and discovered that the T2D susceptibility genes can also be used to estimate the hypoglycemic drug response. We found that paired box 4 (PAX4) and potassium voltage-gated channel subfamily Q member 1 (KCNQ1) can be used to determine the efficacy of repaglinide and rosiglitazone, respectively. Based on the single genetic markers on drug efficacy, we also constructed two genetic scoring models for repaglinide and rosiglitazone drug efficacy, respectively. The model can be used to estimate the responses of different drugs.

**Ethics approval**

Ethical approval was granted by the Institutional Review Board of Shanghai Jiao Tong University Affiliated Sixth People’s Hospital according to Helsinki Declaration II.

### S16 The role of diet in the relationship between environmental pollutants and diabetes risk

#### Qi Sun^1,2^

##### ^1^Channing Division of Network Medicine, Department of Medicine, Brigham and Women’s Hospital and Harvard Medical School, Boston, MA, USA; ^2^Department of Nutrition, Harvard T.H. Chan School of Public Health, Boston, MA, USA

Environmental chemicals that possess endocrine-disrupting properties are known to potentially exert detrimental effects on metabolic health and may lead to an increased risk of developing type 2 diabetes (T2D). The role of diet in the associations between these pollutants and T2D risk has been increasingly recognized. Diet is often the primary source of exposures in general populations without occupational exposure history and thus may serve as confounders for associations between the chemicals and T2D risk. However, in most environmental epidemiological studies, diet was rarely considered in statistical analyses. Moreover, based on the existing knowledge base regarding the biological pathways underlying the effects of diet and the chemicals, it is highly plausible that diet may modulate the effects of chemicals on metabolic health, and the vice versa, although evidence from epidemiological studies is rather sparse for potential interactions between diet and pollutants because of certain methodological challenges. In this lecture, evidence regarding the potentially diabetogenic, food-borne environmental pollutants in relation to diabetes risk is reviewed. Discussions are primarily focus on bisphenol A, phthalates, organic pollutants, perfluoroalkyl substances, and environmental pollutants generated during cooking, such as heterocyclic aromatic amines and polycyclic aromatic hydrocarbons. The role of diet, as primary food sources and confounders, in the associations between these environmental pollutants and T2D risk is explored. Methodological challenges for testing interactions between diet and environmental pollutants are discussed. Furthermore, the potentials of using metabolomics and microbiome as sensitive tools to explore the effects of pollutants on metabolic health, as well as interactions between dietary factors and pollutants are discussed.

**Acknowledgements**

The study was funded by National Institutes of Health (ES021372, ES022981).

**Ethics approval**

All human subjects research conducted by Dr. Sun has been approved by institutional review board committee at the Brigham and Women’s Hospital and Harvard T.H. Chan School of Public Health.

## Session IV Advanced technologies for omics platforms and bioinformatics

### S17 Whole genome sequencing of disease animal models

#### Yixue Li

##### Key Laboratory of Computational Biology, CAS-MPG Partner Institute for Computational Biology, Shanghai Institutes for Biological Sciences, Chinese Academy of Sciences, Shanghai, China

Animal model is always a useful tool for studying fundamental biological questions and clinical translational problems, as said by a Danish Nobel Laureate and a physiologist August Krogh in last century: “For such a large number of problems there will be some animal of choice, or a few such animals, on which it can be most conveniently studied”. Here we presented disease studies based on several special animal models, camel, dog and rabbit as below:

Rapidly evolving genes in the camel lineage are significantly enriched in metabolic pathways, and these changes may underlie the insulin resistance typically observed in these animals. Our analyses may also shed light on the genetic basis of the camel’s remarkable salt tolerance and unusual immune system.

To understand the genetic bases of adaptation to high altitude in dogs, we performed whole-genome sequencing of 60 dogs including five breeds living at continuous altitudes along the Tibetan Plateau from 800 to 5100 m as well as one European breed. More than 15X sequencing coverage for each breed provides us with a comprehensive assessment of the genetic polymorphisms of the dogs, including Tibetan Mastiffs. Comparison of the breeds from different altitudes reveals strong signals of population differentiation at the locus of hypoxia-related genes including endothelial Per-Arnt-Sim (PAS) domain protein 1 (EPAS1) and beta hemoglobin cluster, our results not only indicate parallel evolution of humans and dogs in adaptation to high-altitude hypoxia, but also provide a new opportunity to study the role of EPAS1 in the adaptive processes.

We present the whole-genome sequence of three kinds of the most popular experimental rabbits, NZW, JW and Watanabe heritable hyperlipidemic (WHHL) rabbits and identified 11 new gene mutations possibly associated with the pathophysioloy of WHHL rabbits. We also identified that ALDH2 was significantly down-regulated in the liver of both Chol-fed and WHHL rabbits. These results provide a valuable insight into searching the therapeutic targets of hypercholesterolemia and atherosclerosis.

**Acknowledgements**

This study is supported by the National Key Research and Development Program on Precision Medicine (2016YFC0901700, 2016YFC0902400)

### S18 Revealing dark matter in gene expression by big-data with single-sample network

#### Luonan Chen

##### Key Laboratory of Systems Biology, Shanghai Institutes for Biological Sciences, Chinese Academy of Sciences, Shanghai, China

Generally, a complex disease results not from malfunction of individual molecules but from dysfunction of the relevant system or network, which dynamically changes with time and conditions. Thus, estimating a condition-specific network from a single sample is crucial for elucidating the molecular mechanisms of complex diseases at the system level. However, there is currently no effective way to construct such an individual-specific network by expression profiling of a single sample because of the requirement of multiple samples for computing correlations. We developed statistical methods, i.e. a sample-specific network (SSN) method [1] and Individual-specific edge-network method [2], which allows us to construct individual-specific networks based on molecular expressions of a single sample. Using this method, we can characterize various human diseases at a network level. In particular, such SSNs can lead to the identification of individual-specific disease modules as well as driver genes, even without gene sequencing information. Biological experiments further validated one important advantage of our method over the traditional methods, due to the additional network information. We show that there is rich information for those non-differential genes, not at the gene level but at the network level, which is the dark matter in terms of gene expression, in contrast to the dark matter in terms of gene sequence.

**References**

[1] Liu X, Wang Y, Ji H, et al. Personalized characterization of diseases using sample-specific networks. Nucleic acids research, 2016, 44(22): e164-e164.

[2] Yu X, Zhang J, Sun S, et al. Individual-specific edge-network analysis for disease prediction. Nucleic acids research, 2017, 45(20): e170-e170.

### S19 Databases to cells to genotypes to metabolites – eicosanoid signaling precursors are determined by an ancient polymorphism

#### J. Thomas Brenna

##### Dell Pediatric Research Institute, Department of Pediatrics, Dell Medical School, University of Texas at Austin, Austin, TX, USA

The widespread availability of high throughput methods have enabled rapid advances in establishing associations between single nucleotide polymorphisms and health outcomes. Commonly used single nucleotide polymorphisms (SNPs) were generally chosen to track heritage blocks not connected to causing differences in genetic function via genome-wide association study (GWAS). Diversity in gene function extends beyond SNPs to other genetic elements including insertion-deletions (Indels). Endogenous polyunsaturated fatty acid (PUFA) levels are regulated by diet PUFA. The fatty acid desaturase (FADS) gene cluster has been identified in dozens of GWAS as associated with health and biochemical outcomes. We sought to identify causal variants that control FADS expression and PUFA levels. First computationally we searched the International Hapmap Project database in Japanese participants for expression quantitative trait locus (eQTL) and identified a highly significant region in intron 1 of FADS2 associated with lower expression of FADS1 in the minor allele, and identified predicted consensus biding site for two transcription factors, peroxisome proliferator-activated receptor gamma (PPARg) and sterol regulatory element binding protein (SREBP), and hypothesized that polymorphisms nearby would affect binding. Second lymphoblasts homozygous for the major and minor alleles were cultured leading to the discovery that FADS1 expression is lower in the minor vs major haplotype, and that expression of both FADS1 and FADS2 was strongly modulated by SREBP but not PPARg binding drugs. We sequenced and discovered a 22 bp Indel 137 bp from the SRE: the minor allele is a deletion and the major allele an insertion. Returning to computation with the 1000 genome database we showed that the Indel status causes positive selection related to ancestral diet. Third we compared Indel genotype (rs66698963) from 199 individuals in Kansas City and 1,500 in Beijing against the circulating PUFA and find that circulating arachidonic acid and all omega-3 PUFA are determined genotype. Taken together, these data strongly support rs66698963 Indel as a causal locus in determining arachidonic acid status, with broad implications for inflammation, thrombosis, and related metabolic risk factors. We suggest that genotype at this locus should be considered in all studies of long chain polyunsaturated fatty acid (LCPUFA) interventions.

## Session V Precision nutrition: from research to application and policies

### S20 Precision nutrition concept, product & technology R&D

#### Xuguang Zhang

##### Science and Technology Centre, By-Health Co. Ltd, Guangzhou, China

The United States and Chinese governments have launched “Precision Medicine” projects successively since last one or two years. In 2016, China State Council issued the “Healthy China 2030 Plan Outline”, emphasizing disease prevention as a key national strategy to build a healthy China. In 2017, the role of precision nutrition and technology was stressed for the first time in the Chinese government funded 5-year- “Special Plan (2015-20) of Food Science and Technology Innovation” project, which encourages development of innovative high-techs for producing health foods. More recently, China State Council announced the National Nutrition Program (2017-2030), which addressed the leading roles of advanced nutrition researches and also emphasized application by development of novel technologies and innovative nutritional and healthy foods like nutrient supplementation and nutritional fortified foods to meet different persons’ health needs. Under the guidance of the national policy, the nutrition and health industry ushers in a new period and with numerous opportunities for its development, making “Precision Nutrition” a focus of the industry.

Successful translation from bench-to-bed in application of precision nutrition requires collaborating efforts between research institutes and food and nutrition related industries. Based on the “Open Innovation Platform for joint innovative R&D collaborations”, BY-HEALTH has been planning and developing series of R&D activities relating to “Precision Nutrition”. The product R&D focuses on new function, new raw material and new formulation. For example, BY-HEALTH has launched a product line of Personalised Vitamins, which includes vitamin products targeting at different populations (IVTA) and Vitamin Micro-tablet Project. The technology R&D focuses on precision nutrition including new bio-markers, new medical devices, new evaluating methods, nutrigenomics, health database and intelligent algorithm et al. For example, BY-HEALTH has established a Dried Blood Spot Detection and Evaluation Centre, and launched a Personalised Nutrients Expenditure Evaluation Model in sports nutrition. It collaborates with domestic and international top scientific research institutions to occupy the industrial commanding heights of scientific research, transformation and commercial application platform relating to Precision Nutrition technologies.

**Acknowledgements**

The study was funded by Nutrition Scientific Research Foundation of BY-HEALTH.

### S21 Personalized interventions - a precision approach for the next generation of dietary intervention studies

#### Baukje de Roos

##### The Rowett Institute, University of Aberdeen, Aberdeen, UK

Diet is a key modifiable risk factor for non-communicable diseases. However, we currently are not benefitting from the full potential of its protective effects. Indeed, heterogeneity in the responsiveness to bioactive compounds can obscure associations between their intakes and health outcomes in population based intervention studies. This heterogeneity may partly due to differences between individuals in the absorption, distribution, metabolism and excretion of compounds, which is often the case for, for example, plant bioactives. In addition, physiological and environmental factors may cause heterogeneity in the biological response regarding health outcomes. Identifying the main factors underlying inter-individual differences may help to identify which individuals may particularly benefit from bioactive compounds or dietary patterns.

Nutrition sciences are increasingly adapting approaches from pioneering precision medicine studies in an attempt to overcome and indeed benefit from individualised and/or variable response to therapies. This includes the adaptation of n-of-1 clinical trials that focus on the individual, and not average population response to interventions, and the introduction of multiple, or continuous measurements in individuals over a time course. Aggregated results of many n-of-1 trials offer data on how to better treat sub-populations that share genetic and phenotypic factors, amongst others, or the population at large. A key aspect that will be essential for the development of precision nutrition will be the use of nutrigenomic approaches to allow phenotyping at the individual level. A further important aspect in the delivery of precision nutrition is the development and validation of tools to capture the multidimensional nature of diet. This may include high-throughput nutritional metabolomics complementary to more traditional approaches of assessment of dietary intake and nutrient status.

**Acknowledgements**

The research of Baukje de Roos is supported by the Scottish Government’s Rural and Environment Science and Analytical Services Division (RESAS).

### S22 Exploiting systems flexibility in precision nutrition for health and disease

#### Ben van Ommen

##### TNO, Leiden, The Netherlands

Our (metabolic-inflammatory) health is for a large part determined by the flexibility of multiple processes which collaborate to maintain or regain optimal health. Here, flexibility has a number of nicknames, like robustness, resilience, sensitivity, stress-control and homeostasis. A well-known example is insulin sensitivity, which is an important determinant in glucose homeostasis. Together, these processes shape a system. This is important to realize, as this determines our view on health: all components of the system need to function optimally. In order to optimize this system and regain optimal health, it is essential to quantify the flexibility status of each of the processes, and causally intervene in those processes which perform sub-optimally. In the example of glucose homeostasis, an oral glucose tolerance test can be performed which not only provides insight into the post-challenge response of plasma glucose, but also in the relative contributions of the major processes involved (beta-cell insulin production, and insulin sensitivity of liver, muscle and adipose tissue. For each of these processes, specific nutritional interventions can be designed, making optimizing of glucose control (or curing from type 2 diabetes) a “precision nutrition – precision medicine” combination. I will elaborate on the concepts, mechanisms, quantification methods and intervention options, demonstrating how public health nutrition can evolve into a meaningful precision science and reality, both in daily life and in healthcare.

**Acknowledgements**

Funding of the study came from internal TNO funds.

### S23 Unleashing the full potential of individual nutrients by precision nutrition

#### Michael de Marco

##### BASF SE, Lampertheim, Germany

The consumer is ready for Precision Nutrition and is looking for new solutions for a healthier life. They want to optimize their performance, focus on their individual needs and are aiming to utilize what we call “The quantified self” for this. To serve this consumer need, more and more companies are offering digital health solutions. But how good and beneficial are their offerings? Who will provide a holistic offer to the consumer and whom can I trust as a consumer? Enabling technology is available and ecosystem approaches can combine required technologies, services and products. As a main provider for health ingredients to the market, BASF Newtrition® is willing and already focused on building ecosystems and alliances with other industry partners around the world to provide consumers high quality and trustful ingredients for personalized health solutions. BASF is performing clinical research in-house but also externally to build the most important required foundation for Precision Nutrition: clinical evidence. This presentation will introduce you to some tangible examples and a vision for Precision Nutrition putting the consumer into the center of our efforts.

**Acknowledgements**

The study was funded by BASF SE.

